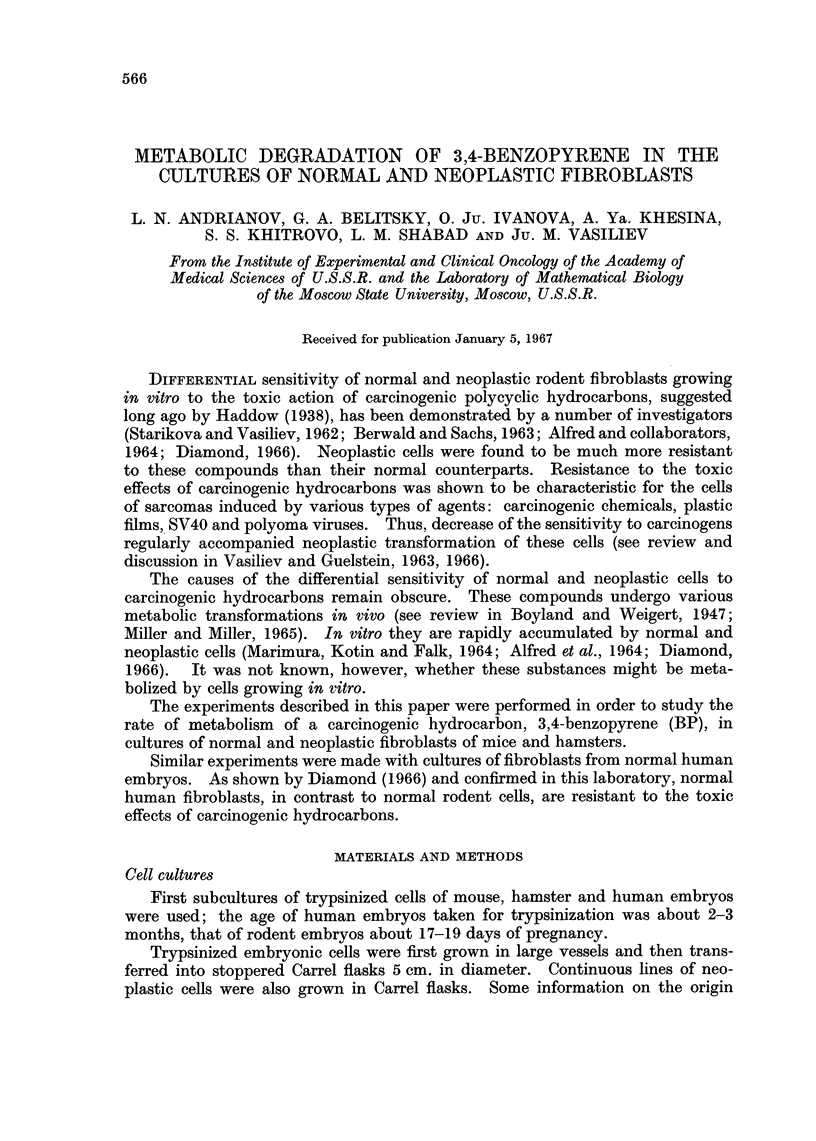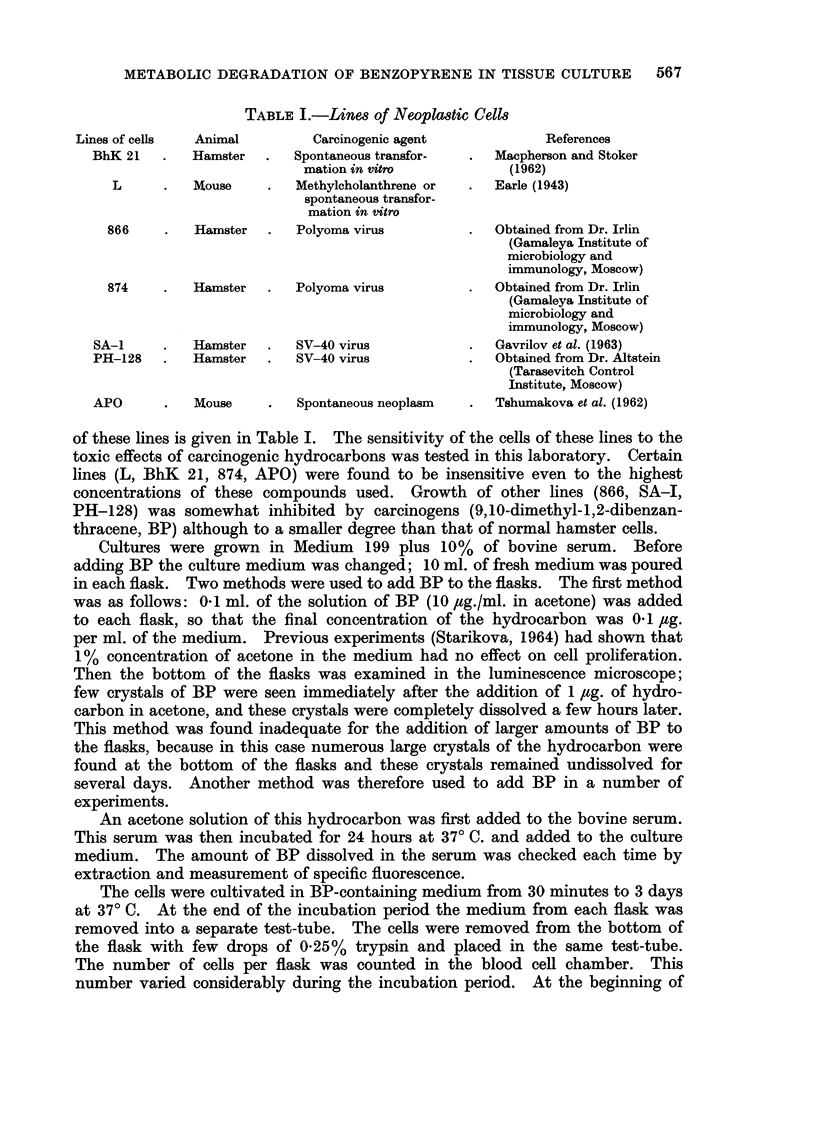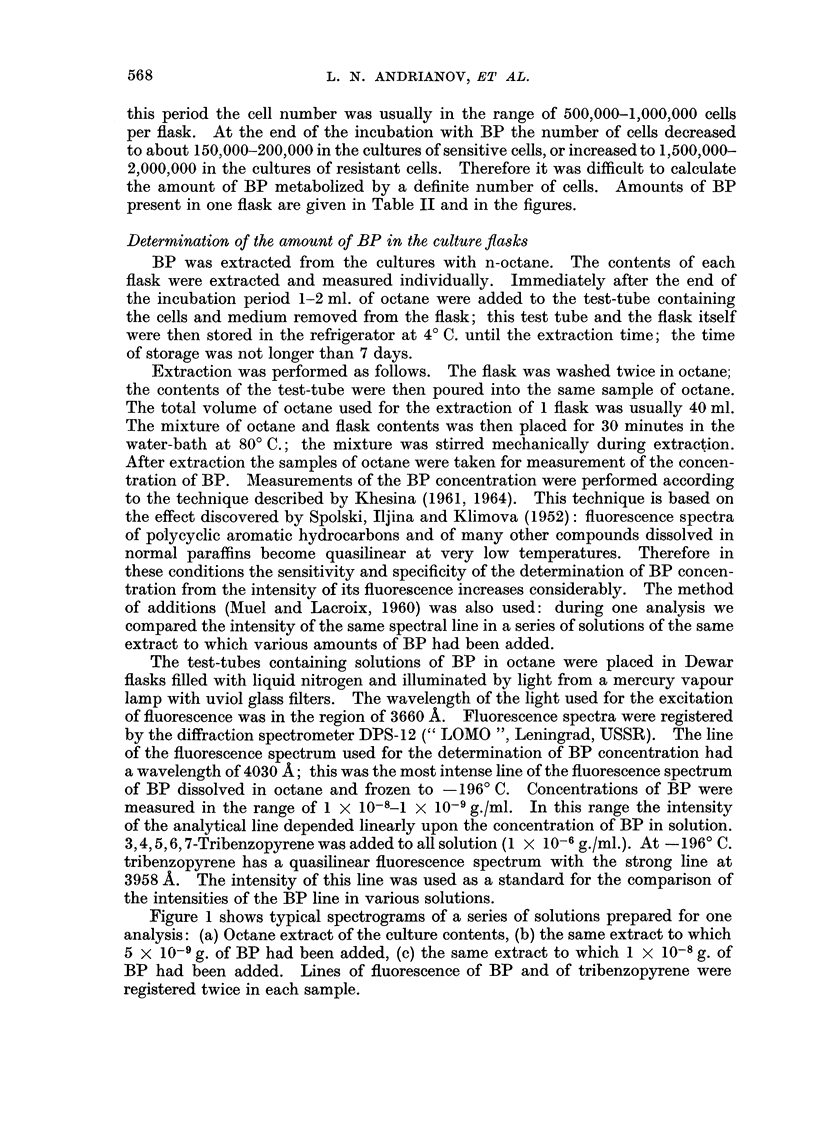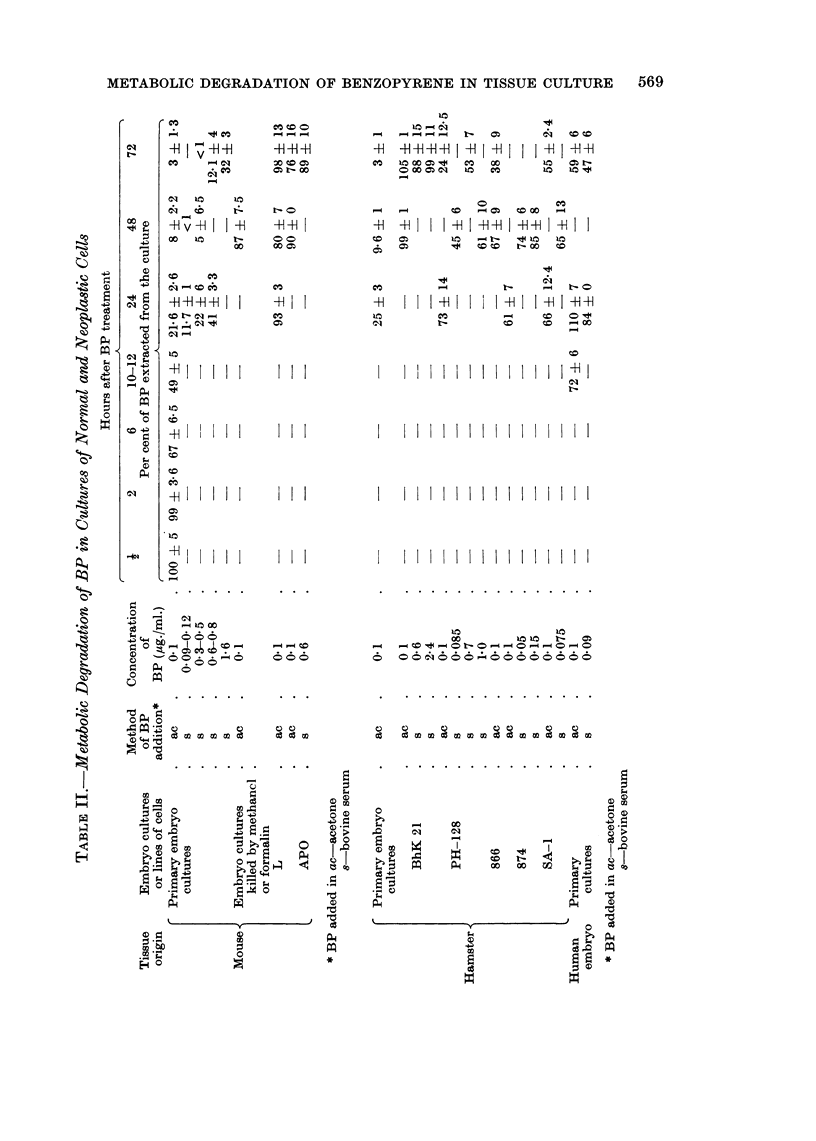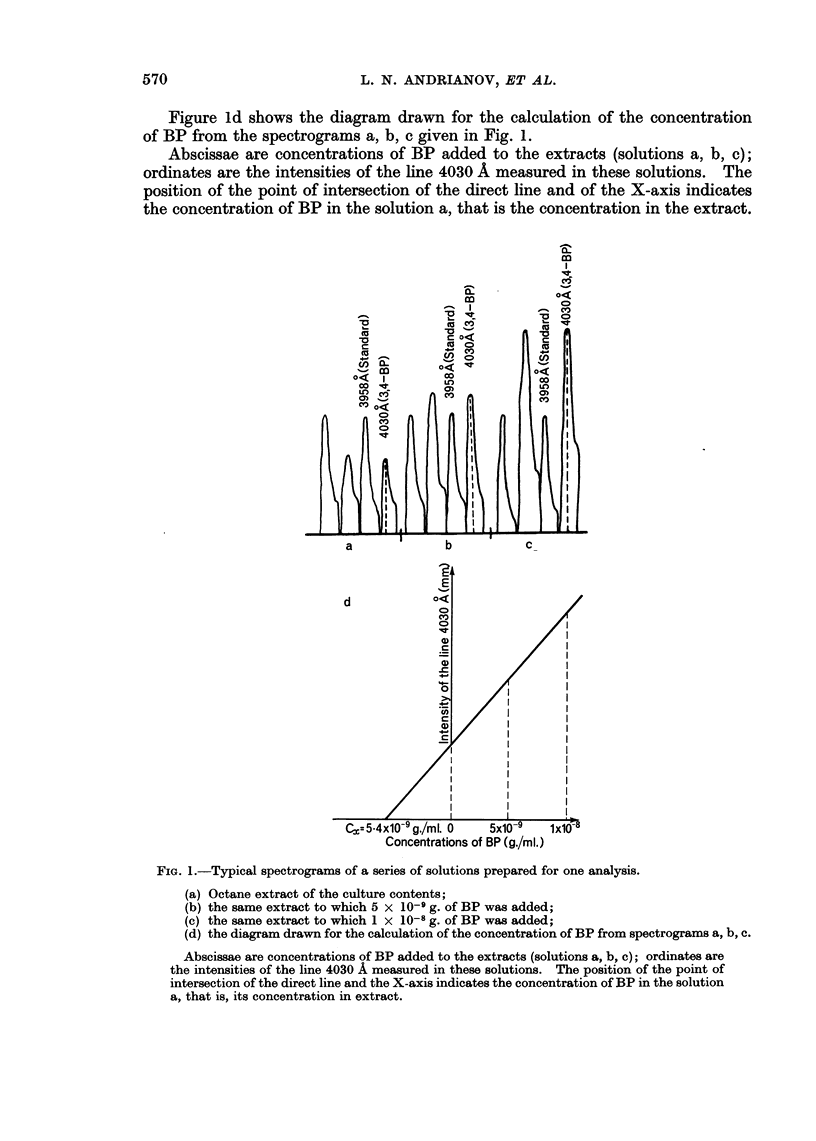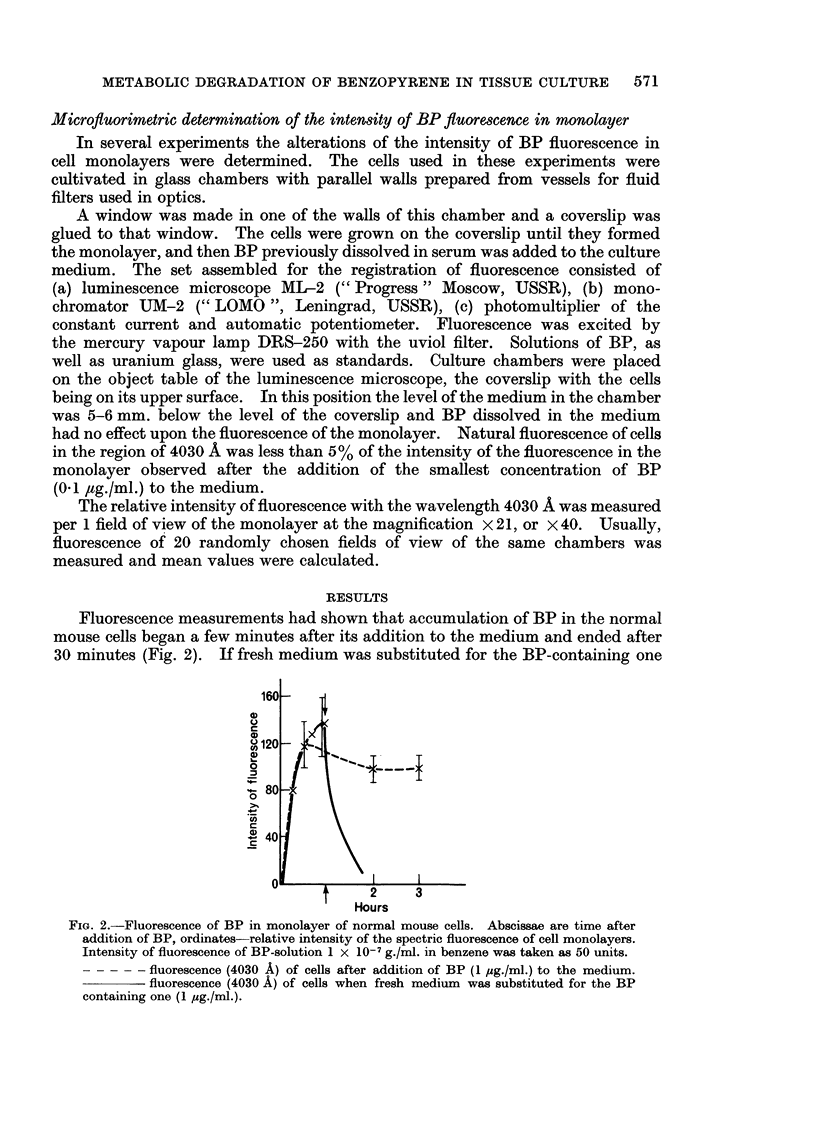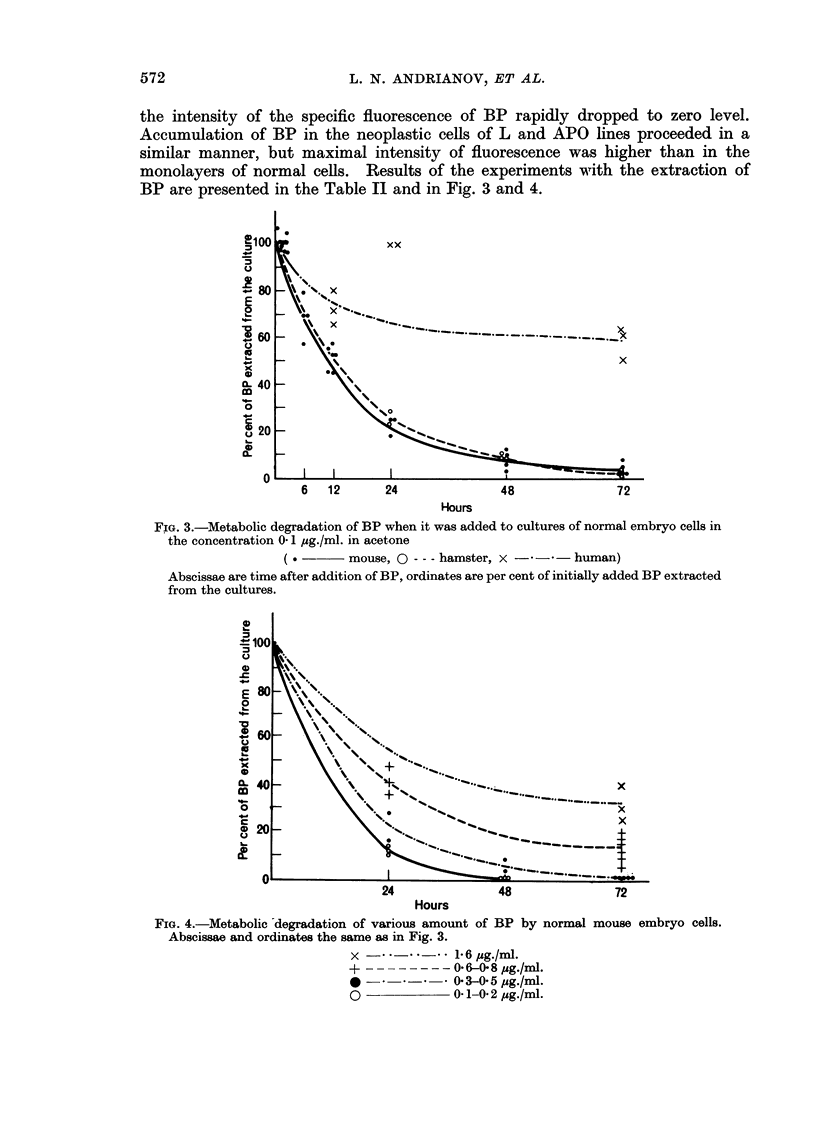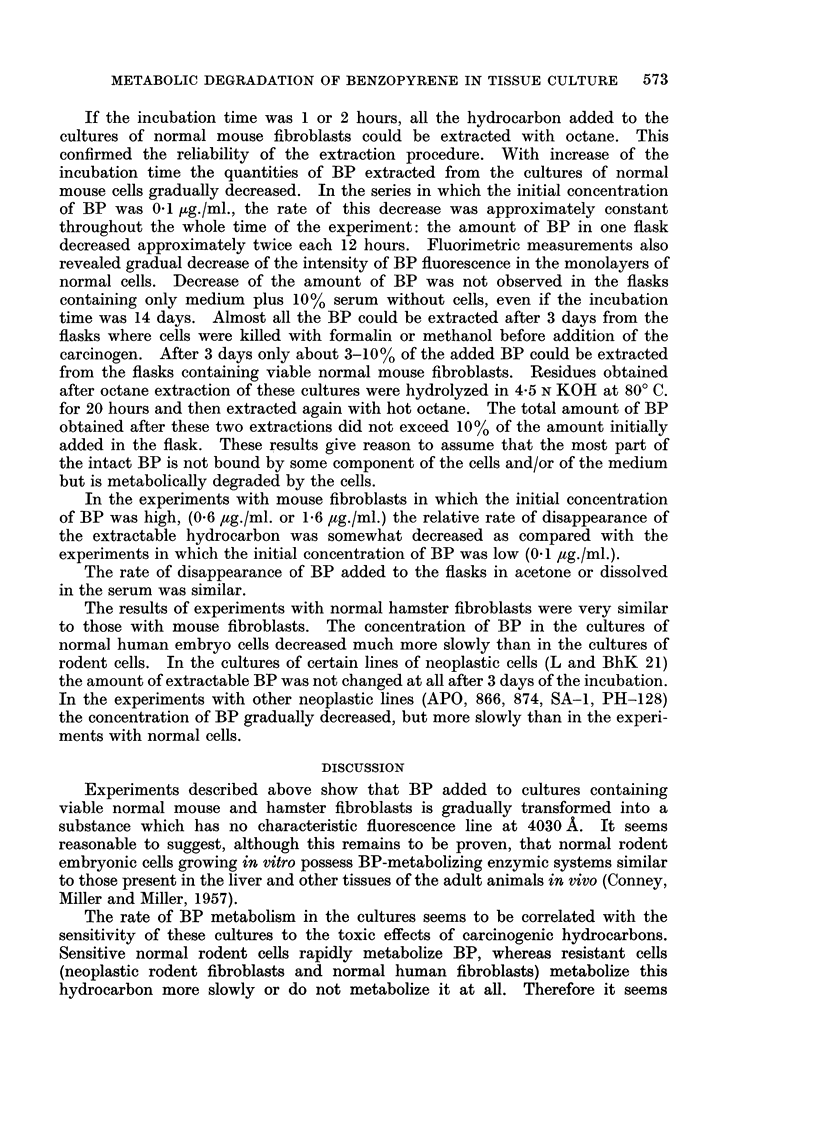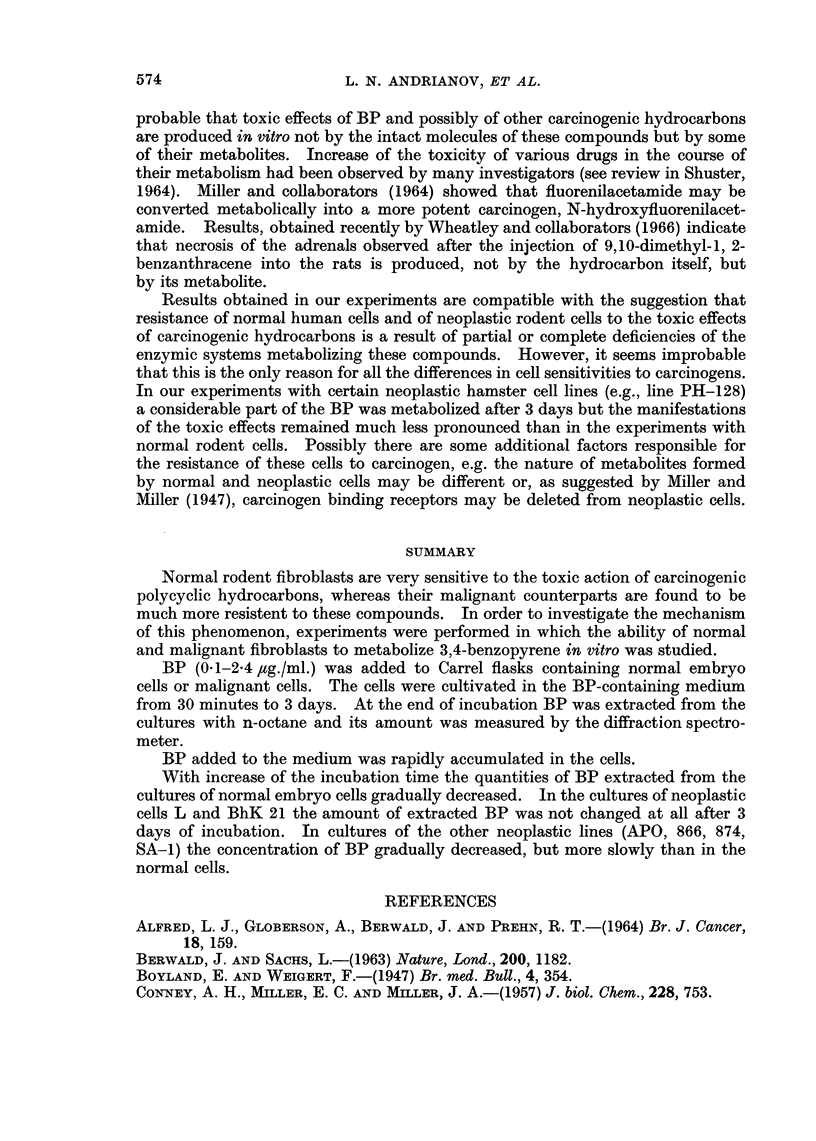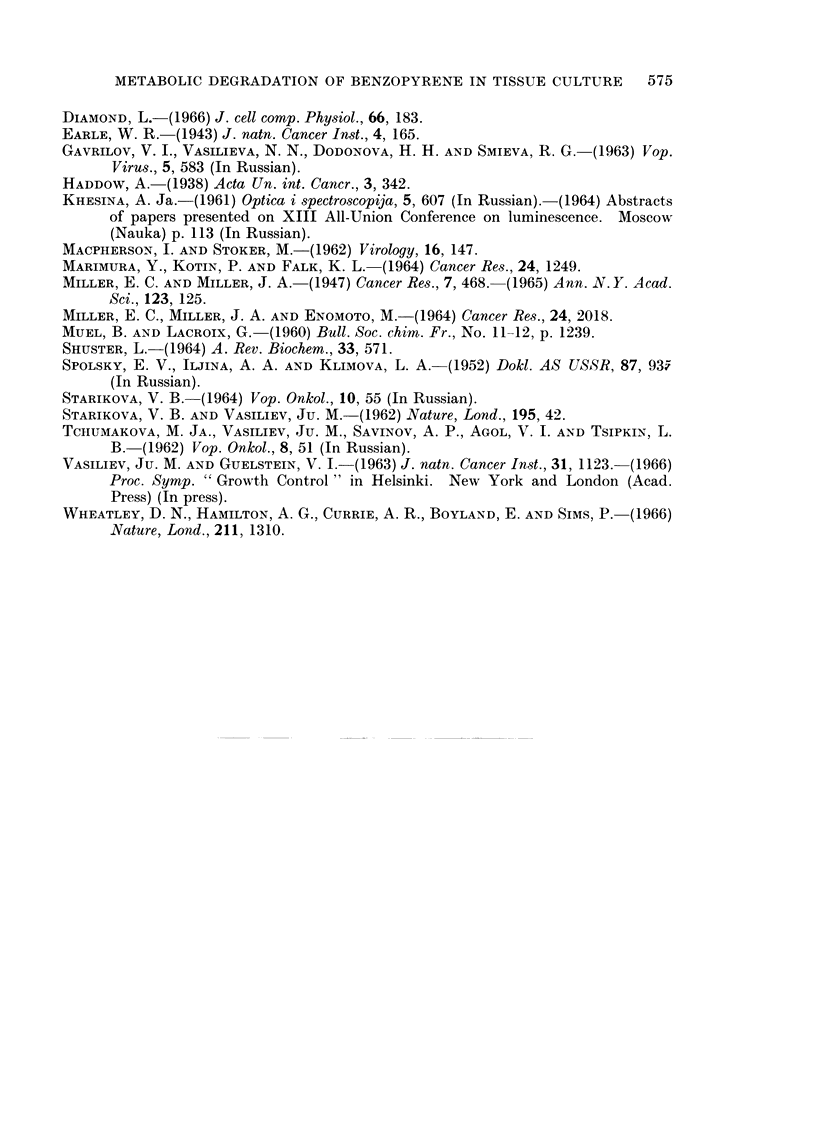# Metabolic degradation of 3,4-benzopyrene in the cultures of normal and neoplastic fibroblasts.

**DOI:** 10.1038/bjc.1967.66

**Published:** 1967-09

**Authors:** L. N. Andrianov, G. A. Belitsky, O. J. Ivanova, A. Y. Khesina, S. S. Khitrovo, L. M. Shabad, J. M. Vasiliev


### METABOLIC DEGRADATION OF 3,4-BENZOPYRENE IN THE CULTURES OF NORMAL AND NEOPLASTIC FIBROBLASTS

L. N. ANDRIANOV, G. A. BELITSKY, O. JU. IVANOVA, A. YA. KHESINA, S. S. KHITROVO, L. M. SHABAD AND JU. M. VASILIEV

*From the Institute of Experimental and Clinical Oncology of the Academy of Medical Sciences of U.S.S.R. and the Laboratory of Mathematical Biology of the Moscow State University, Moscow, U.S.S.R.*

Received for publication January 5, 1967

DIFFERENTIAL sensitivity of normal and neoplastic rodent fibroblasts growing *in vitro* to the toxic action of carcinogenic polycyclic hydrocarbons, suggested long ago by Haddow (1938), has been demonstrated by a number of investigators (Starikova and Vasiliev, 1962; Berwald and Sachs, 1963; Alfred and collaborators, 1964; Diamond, 1966). Neoplastic cells were found to be much more resistant to these compounds than their normal counterparts. Resistance to the toxic effects of carcinogenic hydrocarbons was shown to be characteristic for the cells of sarcomas induced by various types of agents: carcinogenic chemicals, plastic films, SV40 and polyoma viruses. Thus, decrease of the sensitivity to carcinogens regularly accompanied neoplastic transformation of these cells (see review and discussion in Vasiliev and Guelstein, 1963, 1966).

The causes of the differential sensitivity of normal and neoplastic cells to carcinogenic hydrocarbons remain obscure. These compounds undergo various metabolic transformations *in vivo* (see review in Boyland and Weigert, 1947; Miller and Miller, 1965). *In vitro* they are rapidly accumulated by normal and neoplastic cells (Marimura, Kotin and Falk, 1964; Alfred *et al.*, 1964; Diamond, 1966). It was not known, however, whether these substances might be metabolized by cells growing *in vitro*.

The experiments described in this paper were performed in order to study the rate of metabolism of a carcinogenic hydrocarbon, 3,4-benzopyrene (BP), in cultures of normal and neoplastic fibroblasts of mice and hamsters.

Similar experiments were made with cultures of fibroblasts from normal human embryos. As shown by Diamond (1966) and confirmed in this laboratory, normal human fibroblasts, in contrast to normal rodent cells, are resistant to the toxic effects of carcinogenic hydrocarbons.

### MATERIALS AND METHODS

### *Cell cultures*

First subcultures of trypsinized cells of mouse, hamster and human embryos were used; the age of human embryos taken for trypsinization was about 2–3 months, that of rodent embryos about 17–19 days of pregnancy.

Trypsinized embryonic cells were first grown in large vessels and then transferred into stoppered Carrel flasks 5 cm. in diameter. Continuous lines of neoplastic cells were also grown in Carrel flasks. Some information on the origin

TABLE I.—*Lines of Neoplastic Cells*

| Lines of cells | Animal | Carcinogenic agent | References |
|---|---|---|---|
| BhK 21 | Hamster | Spontaneous transformation *in vitro* | Macpherson and Stoker (1962) |
| L | Mouse | Methylcholanthrene or spontaneous transformation *in vitro* | Earle (1943) |
| 866 | Hamster | Polyoma virus | Obtained from Dr. Irlin (Gamaleya Institute of microbiology and immunology, Moscow) |
| 874 | Hamster | Polyoma virus | Obtained from Dr. Irlin (Gamaleya Institute of microbiology and immunology, Moscow) |
| SA–1 | Hamster | SV–40 virus | Gavrilov *et al.* (1963) |
| PH–128 | Hamster | SV–40 virus | Obtained from Dr. Altstein (Tarasevitch Control Institute, Moscow) |
| APO | Mouse | Spontaneous neoplasm | Tshumakova *et al.* (1962) |

of these lines is given in Table I. The sensitivity of the cells of these lines to the toxic effects of carcinogenic hydrocarbons was tested in this laboratory. Certain lines (L, BhK 21, 874, APO) were found to be insensitive even to the highest concentrations of these compounds used. Growth of other lines (866, SA–I, PH–128) was somewhat inhibited by carcinogens (9,10-dimethyl-1,2-dibenzanthracene, BP) although to a smaller degree than that of normal hamster cells.

Cultures were grown in Medium 199 plus 10% of bovine serum. Before adding BP the culture medium was changed; 10 ml. of fresh medium was poured in each flask. Two methods were used to add BP to the flasks. The first method was as follows: 0·1 ml. of the solution of BP (10 $\mu$g./ml. in acetone) was added to each flask, so that the final concentration of the hydrocarbon was 0·1 $\mu$g. per ml. of the medium. Previous experiments (Starikova, 1964) had shown that 1% concentration of acetone in the medium had no effect on cell proliferation. Then the bottom of the flasks was examined in the luminescence microscope; few crystals of BP were seen immediately after the addition of 1 $\mu$g. of hydrocarbon in acetone, and these crystals were completely dissolved a few hours later. This method was found inadequate for the addition of larger amounts of BP to the flasks, because in this case numerous large crystals of the hydrocarbon were found at the bottom of the flasks and these crystals remained undissolved for several days. Another method was therefore used to add BP in a number of experiments.

An acetone solution of this hydrocarbon was first added to the bovine serum. This serum was then incubated for 24 hours at 37° C. and added to the culture medium. The amount of BP dissolved in the serum was checked each time by extraction and measurement of specific fluorescence.

The cells were cultivated in BP-containing medium from 30 minutes to 3 days at 37° C. At the end of the incubation period the medium from each flask was removed into a separate test-tube. The cells were removed from the bottom of the flask with few drops of 0·25% trypsin and placed in the same test-tube. The number of cells per flask was counted in the blood cell chamber. This number varied considerably during the incubation period. At the beginning of this period the cell number was usually in the range of 500,000–1,000,000 cells per flask. At the end of the incubation with BP the number of cells decreased to about 150,000–200,000 in the cultures of sensitive cells, or increased to 1,500,000–2,000,000 in the cultures of resistant cells. Therefore it was difficult to calculate the amount of BP metabolized by a definite number of cells. Amounts of BP present in one flask are given in Table II and in the figures.

*Determination of the amount of BP in the culture flasks*

BP was extracted from the cultures with n-octane. The contents of each flask were extracted and measured individually. Immediately after the end of the incubation period 1–2 ml. of octane were added to the test-tube containing the cells and medium removed from the flask; this test tube and the flask itself were then stored in the refrigerator at 4° C. until the extraction time; the time of storage was not longer than 7 days.

Extraction was performed as follows. The flask was washed twice in octane; the contents of the test-tube were then poured into the same sample of octane. The total volume of octane used for the extraction of 1 flask was usually 40 ml. The mixture of octane and flask contents was then placed for 30 minutes in the water-bath at 80° C.; the mixture was stirred mechanically during extraction. After extraction the samples of octane were taken for measurement of the concentration of BP. Measurements of the BP concentration were performed according to the technique described by Khesina (1961, 1964). This technique is based on the effect discovered by Spolski, Iljina and Klimova (1952): fluorescence spectra of polycyclic aromatic hydrocarbons and of many other compounds dissolved in normal paraffins become quasilinear at very low temperatures. Therefore in these conditions the sensitivity and specificity of the determination of BP concentration from the intensity of its fluorescence increases considerably. The method of additions (Muel and Lacroix, 1960) was also used: during one analysis we compared the intensity of the same spectral line in a series of solutions of the same extract to which various amounts of BP had been added.

The test-tubes containing solutions of BP in octane were placed in Dewar flasks filled with liquid nitrogen and illuminated by light from a mercury vapour lamp with uviol glass filters. The wavelength of the light used for the excitation of fluorescence was in the region of 3660 Å. Fluorescence spectra were registered by the diffraction spectrometer DPS-12 (" LOMO ", Leningrad, USSR). The line of the fluorescence spectrum used for the determination of BP concentration had a wavelength of 4030 Å; this was the most intense line of the fluorescence spectrum of BP dissolved in octane and frozen to −196° C. Concentrations of BP were measured in the range of $1 \times 10^{-8}$–$1 \times 10^{-9}$ g./ml. In this range the intensity of the analytical line depended linearly upon the concentration of BP in solution. 3,4,5,6,7-Tribenzopyrene was added to all solution ($1 \times 10^{-6}$ g./ml.). At −196° C. tribenzopyrene has a quasilinear fluorescence spectrum with the strong line at 3958 Å. The intensity of this line was used as a standard for the comparison of the intensities of the BP line in various solutions.

Figure 1 shows typical spectrograms of a series of solutions prepared for one analysis: (a) Octane extract of the culture contents, (b) the same extract to which $5 \times 10^{-9}$ g. of BP had been added, (c) the same extract to which $1 \times 10^{-8}$ g. of BP had been added. Lines of fluorescence of BP and of tribenzopyrene were registered twice in each sample.

TABLE II.—*Metabolic Degradation of BP in Cultures of Normal and Neoplastic Cells*

| Tissue origin | Embryo cultures or lines of cells | Method of BP addition* | Concentration of BP (μg./ml.) | Hours after BP treatment: ½ | 2 | 6 | 10–12 | 24 | 48 | 72 |
|---|---|---|---|---|---|---|---|---|---|---|
| | | | | Per cent of BP extracted from the culture | | | | | | |
| Mouse | Primary embryo cultures | ac | 0·1 | 100 ± 5 | 99 ± 3·6 | 67 ± 6·5 | 49 ± 5 | 21·6 ± 2·6 | 8 ± 2·2 | 3 ± 1·3 |
| | | s | 0·09–0·12 | — | — | — | — | 11·7 ± 1 | < 1 | — |
| | | s | 0·3–0·5 | — | — | — | — | 22 ± 6 | 5 ± 6·5 | < 1 |
| | | s | 0·6–0·8 | — | — | — | — | 41 ± 3·3 | — | 12·1 ± 4 |
| | | s | 1·6 | — | — | — | — | — | — | 32 ± 3 |
| | Embryo cultures killed by methancl or formalin | ac | 0·1 | — | — | — | — | — | 87 ± 7·5 | |
| | L | ac | 0·1 | — | — | — | — | 93 ± 3 | 80 ± 7 | 98 ± 13 |
| | | ac | 0·1 | — | — | — | — | — | 90 ± 0 | 76 ± 16 |
| | APO | s | 0·6 | — | — | — | — | — | — | 89 ± 10 |

* BP added in *ac*—acetone

*s*—bovine serum

| Tissue origin | Embryo cultures or lines of cells | Method of BP addition* | Concentration of BP (μg./ml.) | ½ | 2 | 6 | 10–12 | 24 | 48 | 72 |
|---|---|---|---|---|---|---|---|---|---|---|
| Hamster | Primary embryo cultures | ac | 0·1 | — | — | — | — | 25 ± 3 | 9·6 ± 1 | 3 ± 1 |
| | | ac | 0 1 | — | — | — | — | — | 99 ± 1 | 105 ± 1 |
| | BhK 21 | s | 0·6 | — | — | — | — | — | — | 88 ± 15 |
| | | s | 2·4 | — | — | — | — | — | — | 99 ± 11 |
| | | ac | 0·1 | — | — | — | — | 73 ± 14 | — | 24 ± 12·5 |
| | PH–128 | s | 0·085 | — | — | — | — | — | 45 ± 6 | — |
| | | s | 0·7 | — | — | — | — | — | — | 53 ± 7 |
| | | s | 1·0 | — | — | — | — | — | 61 ± 10 | — |
| | 866 | ac | 0·1 | — | — | — | — | — | 67 ± 9 | 38 ± 9 |
| | | ac | 0·1 | — | — | — | — | 61 ± 7 | — | — |
| | 874 | s | 0·05 | — | — | — | — | — | 74 ± 6 | — |
| | | s | 0·15 | — | — | — | — | — | 85 ± 8 | — |
| | SA–1 | ac | 0·1 | — | — | — | — | 66 ± 12·4 | — | 55 ± 2·4 |
| | | s | 0·075 | — | — | — | — | — | 65 ± 13 | — |
| Human embryo | Primary cultures | ac | 0·1 | — | — | — | 72 ± 6 | 110 ± 7 | — | 59 ± 6 |
| | | s | 0·09 | — | — | — | — | 84 ± 0 | — | 47 ± 6 |

* BP added in *ac*—acetone

*s*—bovine serum

Figure 1d shows the diagram drawn for the calculation of the concentration of BP from the spectrograms a, b, c given in Fig. 1.

Abscissae are concentrations of BP added to the extracts (solutions a, b, c); ordinates are the intensities of the line 4030 Å measured in these solutions. The position of the point of intersection of the direct line and of the X-axis indicates the concentration of BP in the solution a, that is the concentration in the extract.

FIG. 1.—Typical spectrograms of a series of solutions prepared for one analysis.

(a) Octane extract of the culture contents;
(b) the same extract to which $5 \times 10^{-9}$ g. of BP was added;
(c) the same extract to which $1 \times 10^{-8}$ g. of BP was added;
(d) the diagram drawn for the calculation of the concentration of BP from spectrograms a, b, c.

Abscissae are concentrations of BP added to the extracts (solutions a, b, c); ordinates are the intensities of the line 4030 Å measured in these solutions. The position of the point of intersection of the direct line and the X-axis indicates the concentration of BP in the solution a, that is, its concentration in extract.

*Microfluorimetric determination of the intensity of BP fluorescence in monolayer*

In several experiments the alterations of the intensity of BP fluorescence in cell monolayers were determined. The cells used in these experiments were cultivated in glass chambers with parallel walls prepared from vessels for fluid filters used in optics.

A window was made in one of the walls of this chamber and a coverslip was glued to that window. The cells were grown on the coverslip until they formed the monolayer, and then BP previously dissolved in serum was added to the culture medium. The set assembled for the registration of fluorescence consisted of (a) luminescence microscope ML–2 (" Progress " Moscow, USSR), (b) monochromator UM–2 (" LOMO ", Leningrad, USSR), (c) photomultiplier of the constant current and automatic potentiometer. Fluorescence was excited by the mercury vapour lamp DRS–250 with the uviol filter. Solutions of BP, as well as uranium glass, were used as standards. Culture chambers were placed on the object table of the luminescence microscope, the coverslip with the cells being on its upper surface. In this position the level of the medium in the chamber was 5–6 mm. below the level of the coverslip and BP dissolved in the medium had no effect upon the fluorescence of the monolayer. Natural fluorescence of cells in the region of 4030 Å was less than 5% of the intensity of the fluorescence in the monolayer observed after the addition of the smallest concentration of BP (0·1 $\mu$g./ml.) to the medium.

The relative intensity of fluorescence with the wavelength 4030 Å was measured per 1 field of view of the monolayer at the magnification ×21, or ×40. Usually, fluorescence of 20 randomly chosen fields of view of the same chambers was measured and mean values were calculated.

### RESULTS

Fluorescence measurements had shown that accumulation of BP in the normal mouse cells began a few minutes after its addition to the medium and ended after 30 minutes (Fig. 2). If fresh medium was substituted for the BP-containing one

Fig. 2.—Fluorescence of BP in monolayer of normal mouse cells. Abscissae are time after addition of BP, ordinates—relative intensity of the spectric fluorescence of cell monolayers. Intensity of fluorescence of BP-solution 1 × $10^{-7}$ g./ml. in benzene was taken as 50 units.

– – – – – fluorescence (4030 Å) of cells after addition of BP (1 $\mu$g./ml.) to the medium.
———— fluorescence (4030 Å) of cells when fresh medium was substituted for the BP containing one (1 $\mu$g./ml.).

the intensity of the specific fluorescence of BP rapidly dropped to zero level. Accumulation of BP in the neoplastic cells of L and APO lines proceeded in a similar manner, but maximal intensity of fluorescence was higher than in the monolayers of normal cells. Results of the experiments with the extraction of BP are presented in the Table II and in Fig. 3 and 4.

FIG. 3.—Metabolic degradation of BP when it was added to cultures of normal embryo cells in the concentration 0·1 μg./ml. in acetone

( • ——— mouse, ○ - - - hamster, × —·—·— human)

Abscissae are time after addition of BP, ordinates are per cent of initially added BP extracted from the cultures.

FIG. 4.—Metabolic degradation of various amount of BP by normal mouse embryo cells. Abscissae and ordinates the same as in Fig. 3.

× —··—··—·· 1·6 μg./ml.
\+ - - - - - - - - 0·6–0·8 μg./ml.
● —·—·—·— 0·3–0·5 μg./ml.
○ ——————— 0·1–0·2 μg./ml.

If the incubation time was 1 or 2 hours, all the hydrocarbon added to the cultures of normal mouse fibroblasts could be extracted with octane. This confirmed the reliability of the extraction procedure. With increase of the incubation time the quantities of BP extracted from the cultures of normal mouse cells gradually decreased. In the series in which the initial concentration of BP was 0·1 $\mu$g./ml., the rate of this decrease was approximately constant throughout the whole time of the experiment: the amount of BP in one flask decreased approximately twice each 12 hours. Fluorimetric measurements also revealed gradual decrease of the intensity of BP fluorescence in the monolayers of normal cells. Decrease of the amount of BP was not observed in the flasks containing only medium plus 10% serum without cells, even if the incubation time was 14 days. Almost all the BP could be extracted after 3 days from the flasks where cells were killed with formalin or methanol before addition of the carcinogen. After 3 days only about 3–10% of the added BP could be extracted from the flasks containing viable normal mouse fibroblasts. Residues obtained after octane extraction of these cultures were hydrolyzed in 4·5 N KOH at 80° C. for 20 hours and then extracted again with hot octane. The total amount of BP obtained after these two extractions did not exceed 10% of the amount initially added in the flask. These results give reason to assume that the most part of the intact BP is not bound by some component of the cells and/or of the medium but is metabolically degraded by the cells.

In the experiments with mouse fibroblasts in which the initial concentration of BP was high, (0·6 $\mu$g./ml. or 1·6 $\mu$g./ml.) the relative rate of disappearance of the extractable hydrocarbon was somewhat decreased as compared with the experiments in which the initial concentration of BP was low (0·1 $\mu$g./ml.).

The rate of disappearance of BP added to the flasks in acetone or dissolved in the serum was similar.

The results of experiments with normal hamster fibroblasts were very similar to those with mouse fibroblasts. The concentration of BP in the cultures of normal human embryo cells decreased much more slowly than in the cultures of rodent cells. In the cultures of certain lines of neoplastic cells (L and BhK 21) the amount of extractable BP was not changed at all after 3 days of the incubation. In the experiments with other neoplastic lines (APO, 866, 874, SA–1, PH–128) the concentration of BP gradually decreased, but more slowly than in the experiments with normal cells.

### DISCUSSION

Experiments described above show that BP added to cultures containing viable normal mouse and hamster fibroblasts is gradually transformed into a substance which has no characteristic fluorescence line at 4030 Å. It seems reasonable to suggest, although this remains to be proven, that normal rodent embryonic cells growing *in vitro* possess BP-metabolizing enzymic systems similar to those present in the liver and other tissues of the adult animals *in vivo* (Conney, Miller and Miller, 1957).

The rate of BP metabolism in the cultures seems to be correlated with the sensitivity of these cultures to the toxic effects of carcinogenic hydrocarbons. Sensitive normal rodent cells rapidly metabolize BP, whereas resistant cells (neoplastic rodent fibroblasts and normal human fibroblasts) metabolize this hydrocarbon more slowly or do not metabolize it at all. Therefore it seems probable that toxic effects of BP and possibly of other carcinogenic hydrocarbons are produced *in vitro* not by the intact molecules of these compounds but by some of their metabolites. Increase of the toxicity of various drugs in the course of their metabolism had been observed by many investigators (see review in Shuster, 1964). Miller and collaborators (1964) showed that fluorenilacetamide may be converted metabolically into a more potent carcinogen, N-hydroxyfluorenilacetamide. Results, obtained recently by Wheatley and collaborators (1966) indicate that necrosis of the adrenals observed after the injection of 9,10-dimethyl-1, 2-benzanthracene into the rats is produced, not by the hydrocarbon itself, but by its metabolite.

Results obtained in our experiments are compatible with the suggestion that resistance of normal human cells and of neoplastic rodent cells to the toxic effects of carcinogenic hydrocarbons is a result of partial or complete deficiencies of the enzymic systems metabolizing these compounds. However, it seems improbable that this is the only reason for all the differences in cell sensitivities to carcinogens. In our experiments with certain neoplastic hamster cell lines (e.g., line PH–128) a considerable part of the BP was metabolized after 3 days but the manifestations of the toxic effects remained much less pronounced than in the experiments with normal rodent cells. Possibly there are some additional factors responsible for the resistance of these cells to carcinogen, e.g. the nature of metabolites formed by normal and neoplastic cells may be different or, as suggested by Miller and Miller (1947), carcinogen binding receptors may be deleted from neoplastic cells.

### SUMMARY

Normal rodent fibroblasts are very sensitive to the toxic action of carcinogenic polycyclic hydrocarbons, whereas their malignant counterparts are found to be much more resistent to these compounds. In order to investigate the mechanism of this phenomenon, experiments were performed in which the ability of normal and malignant fibroblasts to metabolize 3,4-benzopyrene *in vitro* was studied.

BP (0·1–2·4 $\mu$g./ml.) was added to Carrel flasks containing normal embryo cells or malignant cells. The cells were cultivated in the BP-containing medium from 30 minutes to 3 days. At the end of incubation BP was extracted from the cultures with n-octane and its amount was measured by the diffraction spectrometer.

BP added to the medium was rapidly accumulated in the cells.

With increase of the incubation time the quantities of BP extracted from the cultures of normal embryo cells gradually decreased. In the cultures of neoplastic cells L and BhK 21 the amount of extracted BP was not changed at all after 3 days of incubation. In cultures of the other neoplastic lines (APO, 866, 874, SA–1) the concentration of BP gradually decreased, but more slowly than in the normal cells.

### REFERENCES

Alfred, L. J., Globerson, A., Berwald, J. and Prehn, R. T.—(1964) *Br. J. Cancer*, **18**, 159.

Berwald, J. and Sachs, L.—(1963) *Nature, Lond.*, **200**, 1182.

Boyland, E. and Weigert, F.—(1947) *Br. med. Bull.*, **4**, 354.

Conney, A. H., Miller, E. C. and Miller, J. A.—(1957) *J. biol. Chem.*, **228**, 753.

DIAMOND, L.—(1966) *J. cell comp. Physiol.*, **66**, 183.
EARLE, W. R.—(1943) *J. natn. Cancer Inst.*, **4**, 165.
GAVRILOV, V. I., VASILIEVA, N. N., DODONOVA, H. H. AND SMIEVA, R. G.—(1963) *Vop. Virus.*, **5**, 583 (In Russian).
HADDOW, A.—(1938) *Acta Un. int. Cancr.*, **3**, 342.
KHESINA, A. Ja.—(1961) *Optica i spectroscopija*, **5**, 607 (In Russian).—(1964) Abstracts of papers presented on XIII All-Union Conference on luminescence. Moscow (Nauka) p. 113 (In Russian).
MACPHERSON, I. AND STOKER, M.—(1962) *Virology*, **16**, 147.
MARIMURA, Y., KOTIN, P. AND FALK, K. L.—(1964) *Cancer Res.*, **24**, 1249.
MILLER, E. C. AND MILLER, J. A.—(1947) *Cancer Res.*, **7**, 468.—(1965) *Ann. N.Y. Acad. Sci.*, **123**, 125.
MILLER, E. C., MILLER, J. A. AND ENOMOTO, M.—(1964) *Cancer Res.*, **24**, 2018.
MUEL, B. AND LACROIX, G.—(1960) *Bull. Soc. chim. Fr.*, No. 11-12, p. 1239.
SHUSTER, L.—(1964) *A. Rev. Biochem.*, **33**, 571.
SPOLSKY, E. V., ILJINA, A. A. AND KLIMOVA, L. A.—(1952) *Dokl. AS USSR*, **87**, 937 (In Russian).
STARIKOVA, V. B.—(1964) *Vop. Onkol.*, **10**, 55 (In Russian).
STARIKOVA, V. B. AND VASILIEV, JU. M.—(1962) *Nature, Lond.*, **195**, 42.
TCHUMAKOVA, M. JA., VASILIEV, JU. M., SAVINOV, A. P., AGOL, V. I. AND TSIPKIN, L. B.—(1962) *Vop. Onkol.*, **8**, 51 (In Russian).
VASILIEV, JU. M. AND GUELSTEIN, V. I.—(1963) *J. natn. Cancer Inst.*, **31**, 1123.—(1966) *Proc. Symp.* "Growth Control" in Helsinki. New York and London (Acad. Press) (In press).
WHEATLEY, D. N., HAMILTON, A. G., CURRIE, A. R., BOYLAND, E. AND SIMS, P.—(1966) *Nature, Lond.*, **211**, 1310.